# Perinatal psychiatry and families’ mental health: Evidence from some french graduated and integrated practices

**DOI:** 10.1192/j.eurpsy.2021.77

**Published:** 2021-08-13

**Authors:** A.-L. Sutter-Dallay

**Affiliations:** Univ. Bordeaux, Inserm, Bph, U1219, F-33000 Bordeaux, France And Perinatal Psychiatry Network-university Department Of Child And Adolescent Psychiatry, Bordeaux University and Charles Perrens Hospital, Bordeaux Cedex, France

## Abstract

**Abstract Body:**

The first years of life represent a crucial period for psycho-affective development - the critical first 1000 days - because the events that happen to infants and babies during this period have psychosocial as well as epigenetic repercussions, with potential consequences throughout life and even for generations to come. The interactive circle that will develop between the skills (and/or vulnerabilities) of infants and parents and the interactive features arising from each triad, must be supported by perinatal mental health policies, of which the joint care of parents and infants in perinatal psychiatry is a pivotal element. It is necessary to develop care pathways, with systems integrated into “usual” care that take into account families from the prenatal or even pre-conceptual period to the postnatal period,

Joint care must also be scalable and thus encompass everything from parent-child psychotherapy to joint mother-baby hospitalisation. This intervention will present and discuss an example of a graduated, integrated and coordinated system of care, and will open up the perspective that perinatal clinicians must bear in mind that joint care is above all "a way of doing things", based on the notions of multidisciplinarity and prevention.

**Disclosure:**

No significant relationships.

